# Genomic Variant Analyses in Pyrethroid Resistant and Susceptible Malaria Vector, *Anopheles sinensis*

**DOI:** 10.1534/g3.120.401279

**Published:** 2020-05-18

**Authors:** Xuelian Chang, Daibin Zhong, Xiaoming Wang, Mariangela Bonizzoni, Yiji Li, Guofa Zhou, Liwang Cui, Xing Wei, Guiyun Yan

**Affiliations:** * Anhui Key Laboratory of Infection and Immunity, Bengbu Medical College, Bengbu, Anhui 233000, China; † Program in Public Health, College of Health Sciences, University of California at Irvine, Irvine, California 92697; ‡ Department of Pathogen Biology, Hainan Medical University, Haikou, Hainan 571100, China; § Department of Internal Medicine, Morsani College of Medicine, University of South Florida, Tampa, Florida 33612

**Keywords:** *Anopheles sinensis*, whole genome sequencing, insecticide resistance, genomic variant, copy number variation, polymerase chain reaction-restriction fragment length polymorphism

## Abstract

*Anopheles sinensis* is a major malaria vector in Southeast Asia. Resistance to pyrethroid insecticides in this species has impeded malaria control in the region. Previous studies found that *An. sinensis* populations from Yunnan Province, China were highly resistant to deltamethrin and did not carry mutations in the voltage-gated sodium channel gene that cause knockdown resistance. In this study, we tested the hypothesis that other genomic variants are associated with the resistance phenotype. Using paired-end whole genome sequencing (DNA-seq), we generated 108 Gb of DNA sequence from deltamethrin -resistant and -susceptible mosquito pools with an average coverage of 83.3× depth. Using a stringent filtering method, we identified a total of 916,926 single nucleotide variants (SNVs), including 32,240 non-synonymous mutations. A total of 958 SNVs differed significantly in allele frequency between deltamethrin -resistant and -susceptible mosquitoes. Of these, 43 SNVs were present within 37 genes that code for immunity, detoxification, cuticular, and odorant proteins. A subset of 12 SNVs were randomly selected for genotyping of individual mosquitoes by polymerase chain reaction-restriction fragment length polymorphism (PCR-RFLP) and showed consistent allele frequencies with the pooled DNA-seq derived allele frequencies. In addition, copy number variations (CNVs) were detected in 56 genes, including 33 that contained amplification alleles and 23 that contained deletion alleles in resistant mosquitoes compared to susceptible mosquitoes. The genomic variants described here provide a useful resource for future studies on the genetic mechanism of insecticide resistance in this important malaria vector species.

Malaria is one of the most important vector-borne diseases in Southeast Asia, and the *Anopheles sinensis* mosquito is the predominant malaria vector. Currently, insecticide-treated bed nets (ITNs) and indoor residual spraying (IRS) are the most important means of mosquito vector control in the global strategy for malaria control (WHO 2019). Pyrethroid are the only class of insecticides approved for use on ITNs due to their high toxicity to insects, rapid rate of knockdown, strong mosquito excito-repellency, and low mammalian toxicity (Diabate *et al.* 2002; Hemingway *et al.* 2004; Kaneko 2011). Extensive use of insecticides has resulted in resistance in many vector mosquito species, including malaria vectors (WHO 2012). The evolution and spread of resistance to insecticides have significantly hampered the efficacy of ITN programs (Alout *et al.* 2014; Coetzee and Koekemoer 2013; Srivastava *et al.* 2010). Tools for early detection of insecticide resistance and resistance surveillance are critical to resistance management and to the rational use of insecticides (WHO 2012).

Development of molecular diagnostic tools for insecticide resistance requires knowledge of resistance mechanisms. In vector mosquito species, at least three mechanisms of physiological resistance to pyrethroids are known (WHO 2012): 1) knockdown resistance (*kdr*) caused by point mutations in the pyrethroid target site, the *para* sodium channel gene, 2) biochemical resistance conferred by metabolic detoxification enzymes such as cytochrome P450 monooxygenases, glutathione S-transferases and esterases, and 3) penetration resistance caused by cuticular thickening. *kdr* mutations and their association with the resistant phenotype have been well studied in many vector species, and markers to monitor resistance have been developed (Martinez-Torres *et al.* 1998; Ranson *et al.* 2000). For example, two of the most common mutations at the same position lead to a change of a Leucine to a Phenylalanine (L1014F) or to a Serine (L1014S) in *kdr* gene are known to confer knockdown resistance to pyrethroids and DDT insecticides in *An. gambiae s.l*. (Platt *et al.* 2015; Lynd *et al.* 2018; Reimer *et al.* 2008; Mitchell *et al.* 2014; Chandre *et al.* 1999) in Africa. However, recent studies support the hypothesis that the *kdr* allele is not fully predictive of the resistant phenotype (Weetman and Donnelly 2015; Donnelly *et al.* 2009; Gnanguenon *et al.* 2015; Okorie *et al.* 2015; Chang *et al.* 2014), and resistance is likely caused by multiple genetic factors (Brooke 2008; Toé *et al.* 2015; Zhong *et al.* 2013). Gene copy number variations (CNVs) have been reported as additional mechanisms of insecticide resistance in *Anopheles* mosquitoes (Djogbénou *et al.* 2008; Lucas *et al.* 2019; Weetman *et al.* 2018a). Highly differentiated copy number variations between population samples were also reported from pooled population sequencing in *Drosophila melanogaster* (Schrider *et al.* 2013) as well as *Aedes aegypti* mosquitoes (Matthews *et al.* 2018).

Whole genome sequencing approaches have proven to be a more powerful tool for achieving a holistic understanding of insecticide resistance mechanisms than classic individual gene-based approaches (Toé *et al.* 2015; Zhu *et al.* 2014; Faucon *et al.* 2015; David *et al.* 2014). To investigate whether different genomic variants other than *kdr* are associated with the resistance phenotype, we compared the genome variation between deltamethrin -resistant and -susceptible mosquitoes from Yunnan, China where the *An. sinensis* mosquito populations lack the *kdr* mutations. First, we developed a comprehensive list of genetic variants in the resistant and susceptible pools of *An. sinensis*. Then, we examined a subset of 12 single nucleotide variants (SNVs) in an additional 40 individuals (20 resistant and 20 susceptible) using polymerase chain reaction-restriction fragment length polymorphism (PCR-RFLP) methods to validate the DNA-seq derived allele frequencies. Finally, we examined the CNVs between deltamethrin -resistant and -susceptible mosquitoes.

## Methods And Materials

### Sample collection and bioassay

In May 2012, *An. sinensis* mosquito larvae and pupae were collected from irrigated rice fields using standard 350ml plastic water dippers in Yinjiang County, Yunnan Province, China. Coordinates for the collection site are latitude 24°45’22.7”N and longitude 98°05’16.4”E, and the elevation is ∼850m. Mosquito larvae and pupae were collected in a variety of habitats with a maximum of 5 individuals per breeding habitat across three villages separated by ∼10 km from each other to avoid sampling siblings. This sampling scheme would yield no more than 2 female adults per habitats based on the assumption of larval-to-adult survivorship 60–80% (Afrane *et al.* 2007) and sex ratio of 1:1 (Phasomkusolsil *et al.* 2011). The collected mosquito larvae and pupae were transported to a local laboratory and reared into adults. All adult mosquitoes were identified to species using the published morphological keys of Dong (Dong 2010). Adult mosquitoes were provided with fresh 10% sucrose solution daily. Adults reared from field-collected larvae and pupae were used in insecticide bioassays to minimize the influence of mosquito age and blood feeding history on resistance measurements. After the mosquitoes were identified to species, *An. sinensis* female adult mosquitoes at 3–5 days post emergence were tested for susceptibility to deltamethrin using the standard WHO resistance tube bioassay (WHO 2013). Briefly, twenty-five mosquitoes were exposed to 0.05% deltamethrin-impregnated paper in an upright plastic tube for 1 h, then transferred into holding tubes and fed on 10% sugar solution on cotton wool for 24 hr. After the 24 hr recovery period, the dead (classified as susceptible) and live mosquitoes (classified as resistant) were preserved individually in 1.5ml tubes containing 1.0 ml 95% ethanol for subsequent DNA extraction and molecular identification of species. A total of 200 female mosquitoes in 8 tubes were tested for resistance against deltamethrin with an additional group of 50 individuals in 2 tubes (without insecticide exposure) as the control group. After the resistance bioassay was completed, all of the insecticide-exposed mosquitoes were preserved and used for subsequent DNA extraction, whereas the 50 living mosquitoes in control tubes were killed and discarded.

### Mosquito DNA extraction and whole genome sequencing

Mosquito DNA was extracted from single mosquitoes using the QIAamp DNA Mini Kit (Qiagen Inc. Valencia, CA) according to the manufacturer’s instructions. The extracted DNA was further cleaned and concentrated using DNA Clean & Concentrator (Zymo Research, Irvine, CA). DNA samples were quantified with Quant-iT PicoGreen dsDNA Assay Kit (Life Technologies, Carlsbad, CA) and checked for quality by 1.0% agarose gel electrophoresis. All mosquitoes were genotyped for species identification by *An. sinensis* allele specific PCR (AS-PCR) following a previously published protocol (Joshi *et al.* 2010). High-quality DNA with equal amounts at the same concentration from each of the 20 resistant mosquitoes were pooled and named YLR (resistant). Similarly, equal amounts of high-quality DNA at the same concentration from each of the 20 susceptible mosquitoes were pooled and named YLS (susceptible). The two DNA pools were used to build paired-end libraries for whole genome DNA sequencing and sequenced on the Illumina Genome Analyzer IIx (GAIIx) at the Broad Institute of MIT and Harvard by running two lanes of PE100 sequencing (100-bp paired-end reads) per pool. Bases were called using Illumina software and data outputted as fastq files.

### Validation of SNV allele frequencies determined by pooled DNA sequencing

To validate the allele frequencies determined by pooled DNA sequencing in the two groups, we examined the allele frequencies by genotyping individual mosquitoes from resistant and susceptible groups. Twenty additional individuals were randomly selected from each of the phenotyped resistant and susceptible groups at the same time as those individuals used for pooled sequencing. Polymerase chain reaction-restriction fragment length polymorphism (PCR-RFLP) analysis was performed for the 40 additional phenotyped individuals at 12 SNVs in 12 randomly selected candidate genes. Gene-specific primers were designed using Primer 3 (http://bioinfo.ut.ee/primer3-0.4.0/). In order to guarantee gene-specificity and avoid amplification of multigene families, primers were designed upon target regions which included the SNVs anchoring outside the conserved region. PCR amplifications were performed in a total volume of 20 μl with 5-20 ng genomic DNA from resistant and susceptible individuals, 10 pmol of forward and reverse primers each with SYBR Green PCR Master Mix (Life Technologies, Grand Island, NY) under the following thermocycling conditions: 95° for 3 min, then 35 cycles of 95° for 30 s, 55° for 30 s, 72° for 45 s, and finally 72° for 6 min. 5 μl of PCR products were used for restriction enzyme digestions (Table S1). After digestion, the products were run on a 2% agarose gel, with undigested PCR product as control. The mutation frequencies were calculated based on the agarose gel electrophoresis bands.

### Data analysis

CLC Genomics Workbench 12.0.3 software (CLCbio, Aahus, Denmark, http://www.clcbio.com) was used for data analyses. First, Trimmomatic 0.36 (Bolger *et al.* 2014) was used to remove adapters and perform a sliding window of trimming to discard sequences with a Phred score of less than 30. Then, reads were filtered based on their quality using the NCBI/Sanger or Illumina pipeline function to trim low-quality reads and filter out failed reads in CLC (Leaché *et al.* 2013). The resulting high-quality paired-end reads from YLR pool, YLS pool, and YLR+YLS (combined reads from both pools) were separately mapped to the *An. sinensis* reference genome (VectorBase, www.vectorbase.org: *Anopheles sinensis*, AsinC2) using the default parameters. SNVs were called in the mapped sequencing reads of the YLR+YLS using the ‘Low Frequency Variant Detection’ tool of CLC against the reference genome of *An. sinensis* (AsinC2). The following parameters were used: required significance = 1%, ignore positions with coverage above = 800, ignore broken pairs = yes (broken paired reads defined as one of the two reads shorter than the set length cutoff at 100-bp after quality trimming), minimum coverage = 80 (40 individuals with at least 2 reads for each individual), minimum count = 28, minimum frequency = 35%, base quality filter = yes, neighborhood radius = 5, minimum central quality = 20, minimum neighborhood quality = 15, read direction filter = yes, direction frequency = 5%, forward/reverse balance >0.25. Pyro-error variants in homopolymer regions with a minimum length of 3 and a frequency below 0.8 were removed. Detection of synonymous and non-synonymous polymorphism was completed by the “Amino Acid Changes” tool within the CLC using variant file resulted from CLC and CDS track files extracted from reference genome. The WEGO software was used to display functional classification of Gene Ontology (GO) (Ye *et al.* 2006).

To identify SNVs in coding regions that differ in allele frequency between resistant and susceptible mosquitoes, variant calling of non-synonymous polymorphism sites was performed on the read mapping from YLR pool and YLS pool, respectively, using a track file of known variants (identified above) as input and the “Identify Known Mutations from mappings” tool within the CLC. The parameters used to filter variants were as follows: minimum coverage = 40 (20 individuals with at least 2 reads for each individual, given a minimum sequence depth > 1), detection frequency = 2.5% (1/40 alleles for detection of singletons), ignore broken pairs = yes, ignore non-specific matches = yes, and create individual tracks = yes. The resulting information from each pool was exported from the CLC to Excel format, which included the variant frequency, read count, read coverage, and other statistics of each variant locus in the read mapping of the two pooled samples. Allele frequency estimates were calculated as the fraction of reads carrying the non-reference alleles or read counts divided by read coverages. Fisher’s exact tests were used to examine the differences of variant allele frequencies between resistant and susceptible samples using the SNP_tools package (Chen *et al.* 2009). Frequency distribution histograms of genome-wide variants were produced using JMP Pro 14.0.0 (SAS Institute Inc., Cary, NC). Allele frequencies were considered significantly different between resistant and susceptible samples (hereafter named as differential variant) if the false discovery rate (FDR) adjusted *p*-value < 0.05 (Benjamini and Hochberg 1995). A sample size of 20 individuals (diploid) in each pool could detect differences larger than 30% in allele frequencies as significant, with a power of 80% at confidence interval of 95% (*p* < 0.05, two-tailed) (Cohen 1988). In order to identify polymorphisms most strongly associated with deltamethrin resistance, we further filtered SNVs for the candidate SNVs in which the absolute allele frequency difference was > 35% (Faucon *et al.* 2015). Copy number variation (CNV) detection of coding regions in deltamethrin -resistant mosquitoes was conducted by CLC using the deltamethrin -susceptible mosquitoes as control against the reference genome of *An. sinensis*. The statistic threshold for significance was set at an FDR adjusted *p*-value < 0.05 for multiple testing (Benjamini and Hochberg 1995) with a low coverage cutoff at 40.

### Data availability

The data sets supporting the results of this article are available in the Sequence Read Archive under the accession number SRR830401 and SRR830336 for the Illumina whole genome sequencing of genomic DNA in resistant and susceptible mosquitoes in *An. sinensis*. Supplemental material available at figshare: https://doi.org/10.25387/g3.10315898

## Results

### Mosquito susceptibility bioassay and whole-genome sequencing

Among the 200 female mosquitoes exposed to insecticide for testing of susceptibility to deltamethrin, 132 individuals were identified as resistant and 64 as susceptible to deltamethrin (mortality rate 32.0%). All mosquitoes were identified as *An. sinensis* by the AS-PCR method. A total of 194,546,524 and 192,880,116 paired-end reads were obtained for deltamethrin-resistant (YLR pool) and -susceptible (YLS pool) samples, respectively. Of the total, 183,041,352 (94.09%) and 181,355,527 (94.02%) reads were mapped to the *An. sinensis* reference genome (AsinC2.1) for YLR pool and YLS pool respectively, resulting in a mean coverage depth of 83.7× and 83.0×. When the reads from the two pooled samples were combined, a total of 364,676,689 reads were mapped to the reference genome, resulting in a mean pooled coverage depth of 166.8× and individual genome coverage depth of 2.1× (Table 1).

**Table 1:** Summary of whole-genome sequencing in pyrethroid resistant (YLR pool) and susceptible (YLS pool) *Anopheles sinensis*

	Deltamethrin resistant (YLR)	Deltamethrin susceptible (YLS)	Combined (YLR+YLS)
**Total number of reads**	194,546,524	192,880,116	387,426,640
**Mapped reads**	183,041,352	181,355,527	364,676,689
**% mapped reads**	94.09%	94.02%	94.13%
**Average length of reads in pairs**	163.44	158.84	161.18
**Broken paired reads**	25,068,830	25,987,373	51,043,207
**Number of bases mapped**	18,487,176,552	18,316,908,227	36,832,345,589
**Sequencing depth**	83.7×	83.1×	166.8×

### Single nucleotide variants in Anopheles sinensis genome

A total of 916,926 SNVs were identified from all the sequence reads (YLR pool and YLS pool combined) against the *An. sinensis* reference genome. Of these, the majority of SNVs (884,686, 96.5%) were located within non-coding regions. Only 32,240 variants (3.5%) were located within coding regions and resulted in amino acid changes. Compared to the reference genome, variants at 5,418 sites within coding regions were fixed in allele frequency at 100% in both the resistant and susceptible pool, and so these sites were filtered out. The remaining 26,822 SNVs were used as variant track (target sites) for identifying SNVs from read mapping of YLR pool and YLS pool, respectively.

**Figure 1:**
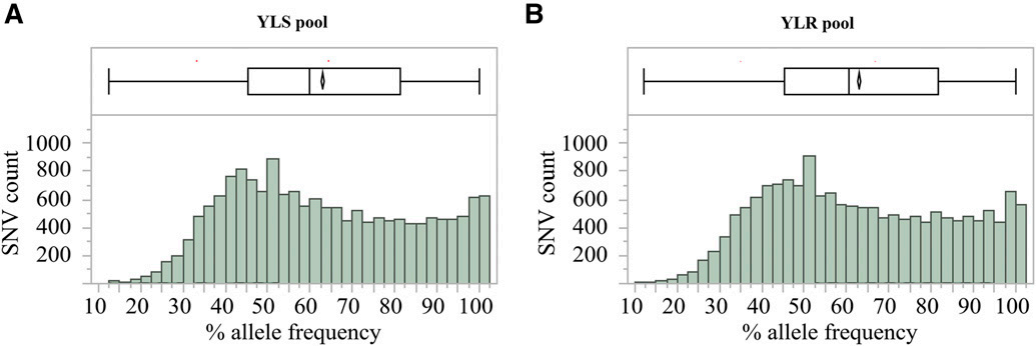
Frequency distribution of single nucleotide variants (SNVs) detected in pyrethroid resistant and susceptible *Anopheles sinensis*. The x-axis represents SNV allele frequency. 0%, all alleles in the pool are reference; 100%, all alleles in the pool are non-reference. The y-axis represents read coverage for SNVs. A: susceptible mosquitoes (YLS pool); B: resistant mosquitoes (YLR pool). In the upper box, 25^th^, 50^th^ and 75^th^ quartiles are displayed; the mean and the 95% confidence interval are represented by a diamond.

**Figure 2:**
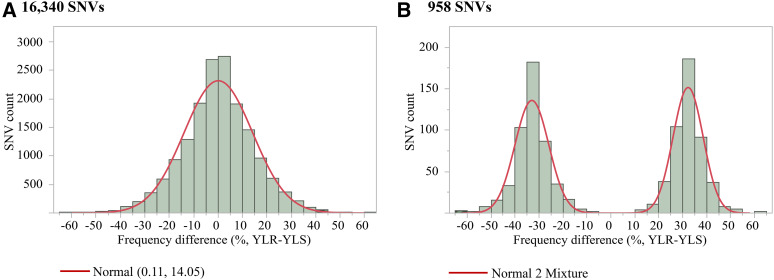
Distribution of allele frequency differences between YLS and YLR pools in *Anopheles sinensis*. A: Distribution of frequency difference between the two pools at 16,340 SNV sites; and B: Distribution of frequency difference between the two pools at the 958 differential SNVs (FDR adjusted *p*-value < 0.05). A negative value indicates high allele frequency in susceptible mosquito pool (YLS), whereas a positive value indicates high allele frequency in resistant mosquito pool (YLR).

**Figure 3:**
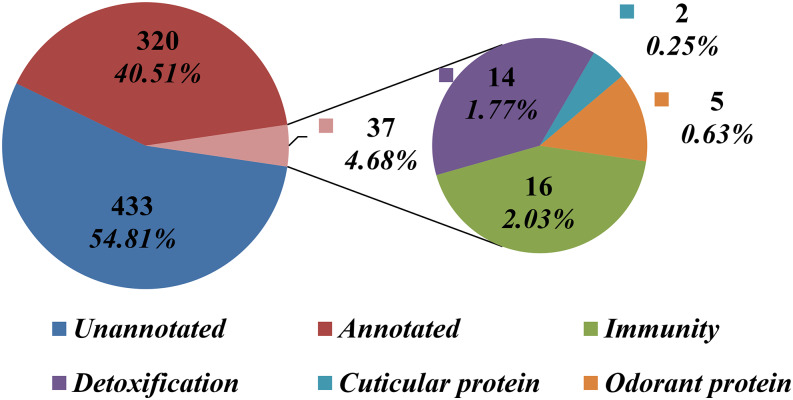
Pie charts show the number and the percentage of genes harboring the differential SNVs detected in pyrethroid resistant and susceptible *Anopheles sinensis*. The function of 37 annotated genes is indicated.

**Figure 4:**
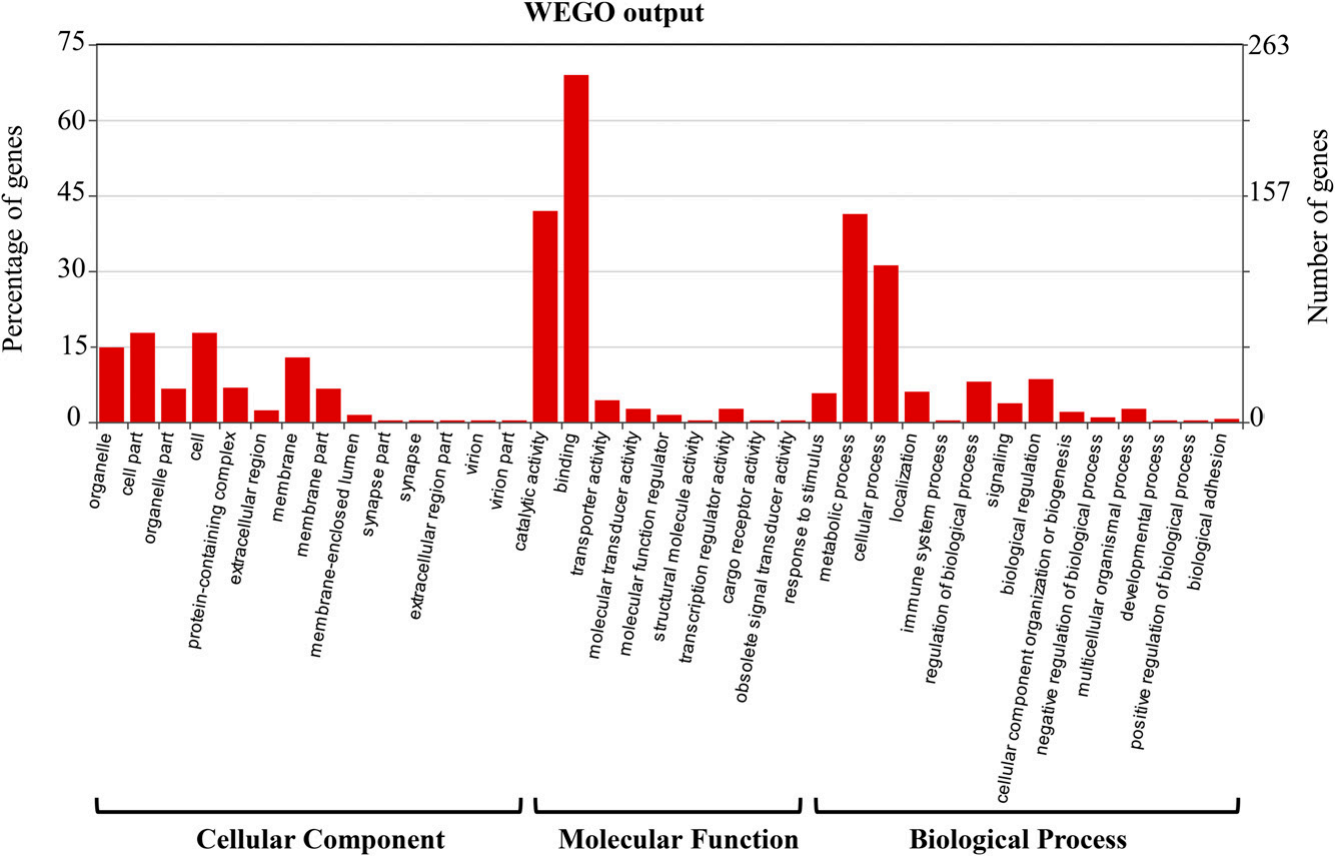
Histogram of Gene Ontology (GO) classification of the 790 genes harboring differential genomic variants in *Anopheles sinensis*. Three main ontologies of GO (biological process, cellular component and molecular function) are shown in the x-axis. The left y-axis indicates the percentage of total genes and the right y-axis is the number of genes in each category.

After we filtered out the low coverage (< 40 reads) SNVs, a total of 16,340 target SNV sites were analyzed for allele frequency differences between the two pools (Table S2). The distributions of variant frequencies in the YLR pool and YLS pool are presented in Figure 1. The frequencies were not distributed normally in both pools (KSL goodness-of-fit test: D = 0.08, *p* < 0.01 for YLS, and D = 0.07, *p* < 0.01 for YLR). An allele frequency comparison of the 16,340 SNVs between YLR pool and YLS pool led to the identification of 958 differential SNVs at an FDR adjusted *p*-value ≤ 0.05 for multiple testing (Figure 2A, Table S2). The distribution of frequency differences between the two pools at 16,340 SNV sites showed the goodness of fit to a normal distribution with a mean of 0.11 and a standard deviation of 14.05 (Figure 2A), whereas the frequency differences of the 958 differential SNVs clearly fit a mixture of two normal distributions (π1 = 0.50, π2 = 0.49; Figure 2B), indicating two groups of samples. The number of SNVs in the two groups (those with higher frequency in resistant and those with lower frequency in resistant pool) were approximately equal. These differential variants were distributed across 790 genes, including immunity, detoxification, cuticular, and odorant proteins at an average mutation rate of 1.6 ± 0.06 per kb (Figure 3, Table S3). More than half of the genes (54.81%, 433/790) were functionally unannotated and ∼40% of these genes were annotated with unknown functions. Among the remaining (4.68%, 37/790) genes of known function, a significant enrichment was detected in the classes of immunity (16) and detoxification proteins (14), followed by odorant proteins (5), and cuticular proteins (2). A total of 43 SNVs were present within these 37 genes. Among the 790 genes with differential variants, 351 were assigned for 1009 GO accession numbers and were classified into 37 function categories under three major domains (biological process, cellular component, and molecular function) (Figure 4, Table S4). Two molecular functions (catalytic activity and binding) and biological processes (metabolic process and cellular process) were highly enriched.

The voltage-gated sodium channel is the target of pyrethroids insecticides. Resistance to pyrethroids is often associated with point mutations in the associated gene, which causes target site insensitivity. After carefully examining the voltage-gated sodium channel gene (GenBank acc. KFB44005, 2138 amino acids), we identified only one non-synonymous mutation (resulting in Ala1072Thr substitute) with a similar allele frequency in deltamethrin -susceptible and -resistant mosquitoes, suggesting that this amino acid change is not related to resistance. The single amino acid change Leu119Phe of glutathione S-transferase gene (GSTe2) has been reported to confer high levels of metabolic resistance to DDT in the malaria vector *An. funestus* (Riveron *et al.* 2014). We examined the variants in this gene in *An. sinensis* (GenBank acc. KFB39338, 221 amino acids) and found two mutations at nucleotide position 355 and 457, resulting in Leu119Val and Ala153Pro amino acid substitutions. However, for both alleles, there was no statistically significant difference in allele frequency between susceptible and resistant mosquitoes, suggesting that this gene is not related to deltamethrin resistance in this population. High organophosphate resistance resulting from insensitive acetylcholinesterase (AChE) by a single mutation (G119S of the *ace-1* gene) has been reported in *Culex pipiens* and in *An. gambiae* (Weill *et al.* 2004). We identified three mutations at positions 239, 269, and 781 (GenBank acc. KFB35326, 385aa), which resulted in Gly80Ala, Val90Ala, and Gly261Ser (corresponding G119S of *ace-1* gene in other species reported) in amino acid substitutions, but with similar allele frequencies in susceptible and resistant mosquitoes. Further study is needed to assess the association between these mutations and organophosphate resistance.

### Candidate genes with the largest difference in allele frequency and high quality

Among the 790 differential genes, 88 (11.1%) genes with high quality (Phred quality score > 35) showed the largest difference (> 40%) in allele frequency between resistant and susceptible pools (FDR adjusted *p*-value < 0.01). These candidates included genes in the classes of immunity (ASIC011903: Met11Ile and ASIC021092: Asn365Asp), detoxification (ASIC012065: Ala1059Thr and ASIC016833: Lys495Thr), and cuticular protein (ASIC010236: Arg25Cys). The candidate genes that contained the 12 most highly differentiated SNVs between YLS and YLR pools are listed in Table 2. Among these genes, only four had gene function descriptions, including two genes (WW domain-binding protein 1 and short-chain dehydrogenase) with a high SNV allele frequency in susceptible mosquitoes and two genes (indolepyruvate ferredoxin oxidoreductase subunit alpha and putative L-carnitine dehydratase/alpha-methylacyl-CoA racemase) with a high SNV allele frequency in resistant mosquitoes.

**Table 2:** List of highly differential SNPs identified between YLS and YLR pools in *Anopheles sinensis*

Transcript ID	GenBank accession	Size (bp)	Gene description	Nucleic acid change	Amino acid change	Allele frequency (%, YLS)	Allele frequency (%, YLR)	Adjusted *p*-value
**YLS > YLR**								
ASIC003949	KFB36789	3897	WW domain-containing protein 1	2644A > G	Thr882Ala	66.7	14.0	6.17E-04
ASIC004261	KFB37064	1236	hypothetical protein ZHAS_00004261	159G > A	Met53Ile	69.6	16.0	2.46E-04
ASIC008461	KFB40921	7764	AGAP002735-PA-like protein	2275C > A	His759Asn	70.8	15.0	2.75E-04
ASIC021070	KFB52802	432	hypothetical protein ZHAS_00021070	283G > T	Gly95Cys	71.4	20.8	5.57E-04
ASIC015363	KFB47421	1479	hypothetical protein ZHAS_00015363	1459T > C	Cys487Arg	77.6	27.5	5.50E-04
ASIC001969	KFB35388	183	short-chain dehydrogenase	43C > T	Arg15Trp	86.4	23.3	1.33E-05
ASIC002752	KFB35824	3462	AGAP002548-PA-like protein	1081T > C	Phe361Leu	86.4	25.6	3.34E-05
**YLR > YLS**								
ASIC009989	KFB42304	2247	hypothetical protein ZHAS_00009989	976C > T	Pro326Ser	23.4	77.5	5.64E-04
ASIC011867	KFB44037	333	indolepyruvate ferredoxin oxidoreductase subunit alpha	252T > G	Asp84Glu	25.0	79.5	4.63E-04
ASIC021208	KFB52925	4215	AGAP005789-PA-like protein	1354T > G	Leu452Val	40.0	100	6.33E-06
ASIC010615	KFB42862	444	AGAP008279-PA-like protein	185C > T	Thr62Met	44.4	95.2	2.74E-04
ASIC003791	KFB36595	492	putative L-carnitine dehydratase/alpha-methylacyl-CoA racemase	280T > A	Ser94Thr	47.6	97.7	2.61E-04

### Relationship between pooled DNA-seq and individually genotyping determined allele frequencies

**Figure 5:**
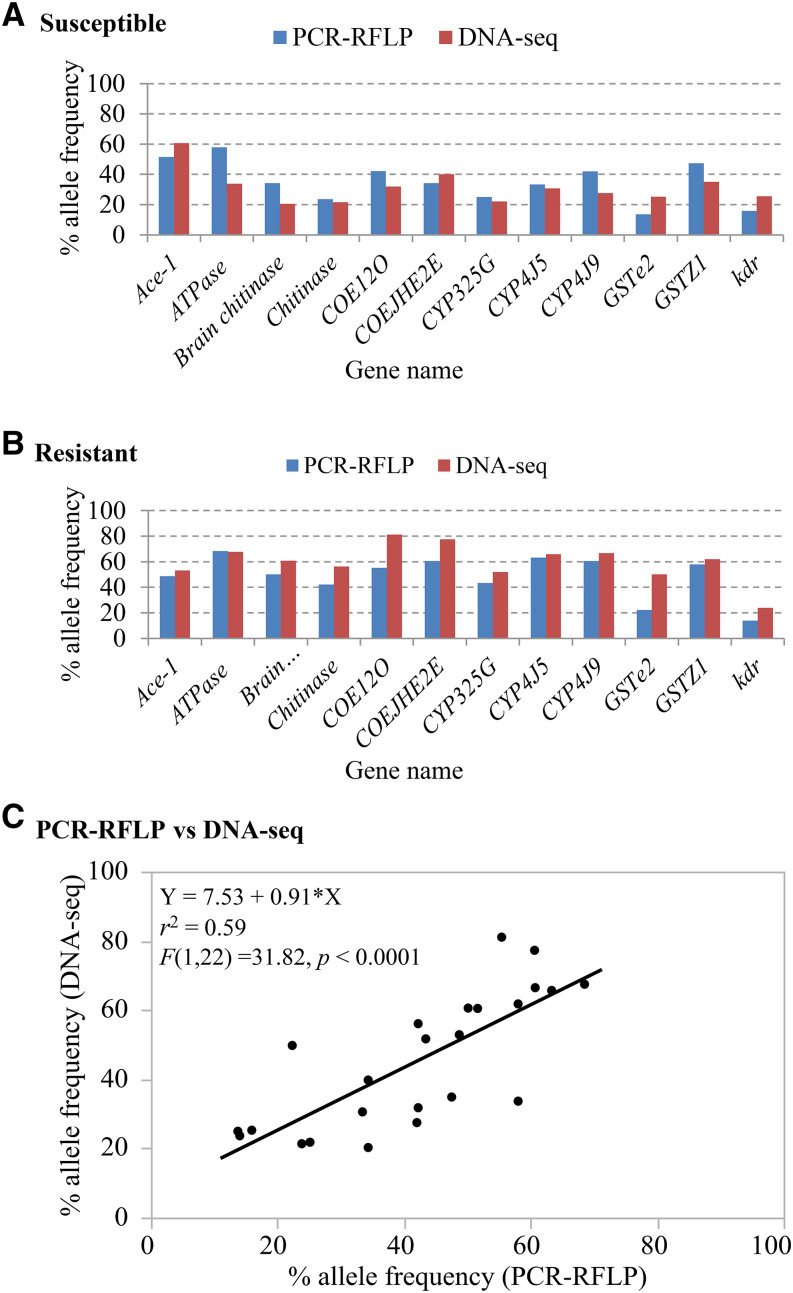
Validation of SNV allele frequencies determined by pooled DNA-seq in pyrethroid susceptible and resistant *Anopheles sinensis* mosquitoes. A: comparison of allele frequencies determined by pooled DNA-seq and individually PCR-RFLP genotyping in susceptible mosquitoes; B: comparison of allele frequencies determined by pooled DNA-seq and individually PCR-RFLP genotyping in resistant mosquitoes; C: relationship between pooled DNA-seq and individually PCR-RFLP genotyping determined allele frequencies. A zero-allele frequency means that all alleles are reference alleles, whereas 100% allele frequency indicates that all allele are non-reference alleles.

To examine the difference between pooled DNA sequencing (DNA-seq) and individually genotyping (PCR-RFLP) determined allele frequencies, an additional 40 phenotyped mosquitoes (20 resistant and 20 susceptible) from the same mosquito population were genotyped individually using PCR-RFLP methods. A list of designed primers, restriction enzymes used for PCR-RFLPs genotyping, and digestion size are shown in Table S1. Similar patterns of allele frequencies and significant correlations were observed between pooled DNA-seq and individually PCR-RFLP genotyping (Figure 5A and 5B), with a simple linear regression coefficient of *r*^2^ = 0.59, *F* (1, 22) = 31.82, and *P* < 0.001 (Figure 5C), suggesting that the allele frequencies were consistent between pooled DNA-seq and individual PCR-RFLP genotyping.

### Copy number variation (CNV) in deltamethrin -resistant and -susceptible mosquitoes

Copy number variations (CNVs) play an important role in evolution and adaptation. CNVs may contribute to insecticide resistance and affect gene structures and expression levels. Out of the 19,708 reference genes (AsinC2) included in the analysis, 56 (0.28%) were detected in copy number variation with fold changes >1.2 at an FDR adjusted *p*-value < 0.001. Of these 56 genes, 33 had increased gene copy numbers (amplification) in resistant mosquitoes, whereas 23 showed loss of gene copy numbers (deletion) in resistant mosquitoes (Table S5). The two genes ASIC004384 (Histidine kinase) and ASIC017541 with increased gene copy numbers showed the highest fold change (1.34), followed by ASIC004581(1.33) and ASIC011293 (1.33) in resistant mosquitoes. The two genes ASIC020925 (Protein PRRC2A isoform X1) and ASIC009866 with decreased gene copy number had the highest fold change (1.44), followed by ASIC006698 (Glutathione s-transferase E2) (1.36) in resistant mosquitoes. The other genes with CNVs included ASIC002830 (Glutaredoxin), ASIC012426 (Glycosyl transferase family 2), ASIC013265 (virulence protein), ASIC015344 (Alcohol dehydrogenase GroES domain protein), as well as 35 genes without known functions. Increased gene copy number of GSTe2 associated with insecticide resistance has been reported in several mosquito species, including *Cx. quinquefasciatus* (Kothera *et al.* 2019), *Aedes aegypti* (Faucon *et al.* 2015), and *An. gambiae* (Lucas *et al.* 2019). Interestingly, we detected a decreased trend of CNVs (fold change = -1.36) of GSTe2 in the resistant mosquitoes, suggesting that this gene might play a different role compared to those *Anopheles* species from Africa (Lucas *et al.* 2019).

## Discussion

In the study, we described the whole-genome sequencing of pooled samples and subsequent identification of genetic variants in the genome of *An. sinensis*, an important malaria vector in Southeast Asia. Identifying genomic variants is a crucial step for unraveling the relationship between genotypes and insecticide resistance phenotypes and can yield important insights into insecticide resistance mechanisms. Our results suggested that resistance to deltamethrin in *An. sinensis* was not caused by the *kdr* mutation as reported in other mosquito species, but the differential SNVs detected in this study could play important roles in deltamethrin resistance in this species. Additionally, the detected gene copy number variations (CNVs) here may also be responsible for deltamethrin resistance. The genetic variants identified in this study represent a significant resource for future investigations into the mechanisms of *An. sinensis* insecticide resistance.

The newly identified genomic variants, such as SNV (Asp84Glu) in indolepyruvate ferredoxin oxidoreductase subunit alpha gene and SNV (Ser94Thr) in L-carnitine dehydratase/alpha-methylacyl-CoA racemase gene, might have some important implications in the role of pyrethroid resistance since they showed significantly higher mutation frequencies in the resistant mosquitoes. In addition, the cytochrome P450 detoxification enzyme genes, such as CYP4J5 (ASIC012923: Thr386Lys and Glu162Asp), CYP9J4 (ASIC017824: Val299Ala), and COEJHE5E(ASIC016833: Lys495Thr) were also detected with significantly higher mutation frequencies in the resistant mosquitoes, suggesting they have a role in pyrethroid insecticide resistance. Resistance-associated SNVs in P450 genes were also detected in other vector mosquito species, including *An. gambiae* (Weetman *et al.* 2018b), *An. arabiensis* (Lynd *et al.* 2019), and *An. funestus* (Tchigossou *et al.* 2018). Overexpression of CYP9J4 gene linked to insecticide resistance were found in *An. gambiae* (Nkya *et al.* 2014) and *An. arabiensis* (Matowo *et al.* 2014). Furthermore, several genes with unknown functions showed highly differential genomic variants. Further studies are needed to illustrate their roles in insecticide resistance.

This study demonstrated that pooled DNA-seq is a cost-effective and powerful tool for analysis of genome-wide allele frequency data in deltamethrin -resistant and -susceptible *An. sinensis* mosquitoes. However, there are also some limitations with the techniques used in this study. A pooled DNA-seq method is prone to alignment problems due to copy number variation or problems in reference genomes (Schlötterer *et al.* 2014). In addition, our sample size of 20 individuals for each pool resulting sequencing depth per individual of 2.1× was relatively small. Therefore, the detection of low-frequency alleles was challenging due to the difficulty in distinguishing them from sequencing errors. The pool size coupled with the amount of sequence generated could limit the detection of rare SNVs. It was only possible to detect the SNVs with 10% or higher frequency in this study. Furthermore, the technical errors in pipetting or DNA quantification may result in imbalanced pools affecting the results (Sham *et al.* 2002).

In summary, this was the first study describing genome variation in *An. sinensis* mosquitoes and comparing mutations between insecticide -resistant and -susceptible *An. sinensis* populations. We identified over 30 thousand non-synonymous variants with nearly one thousand differentiating SNVs as well as 56 CNVs between deltamethrin- resistant and -susceptible populations. Of these, 37 genes with function annotations belonged to the classes of immunity, detoxification, cuticular protein, and odorant protein. The genomic variations and copy number variations described here provided a useful resource for future studies of insecticide resistance mechanisms.
